# Dietary Considerations in Cholecystectomy: Investigating the Impact of Various Dietary Factors on Symptoms and Outcomes

**DOI:** 10.7759/cureus.61183

**Published:** 2024-05-27

**Authors:** Tushar Dahmiwal, Anup Zade, Darshana Tote, Srinivasa Reddy, Kesav Sudabattula

**Affiliations:** 1 General Surgery, Jawaharlal Nehru Medical College, Datta Meghe Institute of Higher Education and Research, Wardha, IND

**Keywords:** post-cholecystectomy syndrome, gall bladder, high fat, diet, cholecystectomy

## Abstract

Cholecystectomy is commonly performed to address gallstone diseases, including the development of gallstones, which can lead to symptoms such as nausea, vomiting, and abdominal pain. Bile acids (BAs) produced by the liver are primarily stored and concentrated in the gallbladder (GB). After cholecystectomy, the body's ability to digest lipids is reduced due to the absence of the GB. Post-cholecystectomy syndrome (PCS) can occur when abdominal symptoms manifest after surgery. The purpose of this review is to look at the various effects of different dietary factors on patients undergoing cholecystectomy, how they affect their overall health after surgery, and how they contribute to symptoms of PCS. Some individuals may experience mild discomfort or alterations in bowel patterns, especially after consuming high-fat meals. The findings from the conducted studies suggest that, although dietary changes are a common recommendation, these measures are not sufficiently supported by evidence when it comes to alleviating symptoms and improving outcomes post-cholecystectomy. The studies found that subjects who consumed particular foods, such as processed meat and fried fatty foods, had exacerbated symptoms after cholecystectomy. Further studies are still required to understand the precise food factors that might affect post-surgical symptoms, as well as outcomes, and to develop tailored measures to enhance patient care and long-term prognosis after undergoing cholecystectomy.

## Introduction and background

A cholecystectomy is a surgical procedure that involves the removal of the gallbladder (GB). The GB, a small organ located beneath the liver, primarily stores bile, a digestive fluid produced by the liver. To aid in the digestion of fats, the GB releases bile into the small intestine. Cholecystectomy is commonly used to address GB disease, including the development of gallstones, which can lead to symptoms such as nausea, vomiting, and abdominal pain. Bile acids (BAs) produced by the liver are primarily stored and concentrated in the GB [1]. Cholecystokinins released by the duodenum trigger GB contractions to release bile during fasting. Bile salts and BAs aid in the absorption of dietary lipids. Changes in the metabolic balance of BA post-cholecystectomy can result in increased reabsorption of BA and enterohepatic circulation [2,3]. Epidemiological studies indicate that GB removal raises the risk of colorectal cancer (CRC) and non-alcoholic fatty liver disease [4].

Gallstones blocking the bile ducts are a common culprit behind cholecystitis, an inflammation of the GB. The buildup of bile in the GB due to obstruction by gallstones triggers inflammation and swelling. Cholecystitis can also stem from blockages in the cystic or bile ducts, in addition to gallstones. Tumors, narrowing of the bile ducts (strictures), or pressure from surrounding structures can all be contributing factors in these blockages. In some cases, cholecystitis might be caused by a GB infection. When bile becomes stagnant due to blockage, it can lead to inflammation and infection, potentially resulting in bacterial infections. Gallstones may or may not be present in such instances [5-7].

There are two primary types of cholecystectomy: laparoscopic and open cholecystectomy. In a minimally invasive laparoscopic cholecystectomy, small abdominal incisions are made. A laparoscope, which is a tiny, flexible tube with a light and camera, is then inserted through a single incision, allowing the surgeon to view the area on a monitor [8]. Following this, small incisions are made, and specialized instruments are utilized to remove the GB. On the other hand, open cholecystectomy is a traditional, open procedure that involves a wider incision in the abdominal wall. Nowadays, open cholecystectomy is less common and is typically reserved for cases in which laparoscopic surgery is not feasible or when complications occur [9,10]. After a cholecystectomy, the body's ability to digest lipids is somewhat reduced due to the absence of the GB. Some individuals may experience mild discomfort or alterations in bowel patterns, especially after consuming high-fat meals. Nevertheless, most people can lead normal and healthy lives even without a GB [11-13].

Post-cholecystectomy syndrome (PCS) can occur when abdominal symptoms manifest after surgery. PCS has been reported to affect 5% to 40% of individuals following cholecystectomy. Symptoms may encompass an upset stomach, nausea, vomiting, gas, bloating, diarrhea, or persistent pain in the upper right abdomen [14]. It is believed that these symptoms are not caused by, but rather exacerbated by the cholecystectomy. Furthermore, patients may also experience symptoms of gastritis as a result of duodenal-gastric reflux of BAs. This reflux may also contribute to the symptoms of PCS. After cholecystectomy, there is no universal standard for medical nutrition therapy (MNT) [15]. MNT should be tailored to the individual patient's needs, and various dietary adjustments may be necessary. It is recommended to restrict fat intake for a few months to allow the liver to adjust to the absence of the GB. Subsequently, fat reintroduction should be gradual, and excessive amounts at any single meal should be avoided [14].

Objective of the study

A review of the available literature is required to fully understand the effect of various dietary factors in cholecystectomy cases, though there is less literature on this topic. The purpose of this review is to investigate the various effects of different dietary factors on patients undergoing cholecystectomy, how they affect their overall health after surgery, and how they contribute to symptoms of PCS. This review provides an overview of how dietary changes can affect patients and what modifications can be made to improve the outcome. This review will examine the existing literature to identify potential dietary factors in cholecystectomy patients, such as the various post-operative symptoms, effects on biochemical factors, caloric intake proportions, and pre-operative impacts.

## Review

Methodology

**Figure 1:**
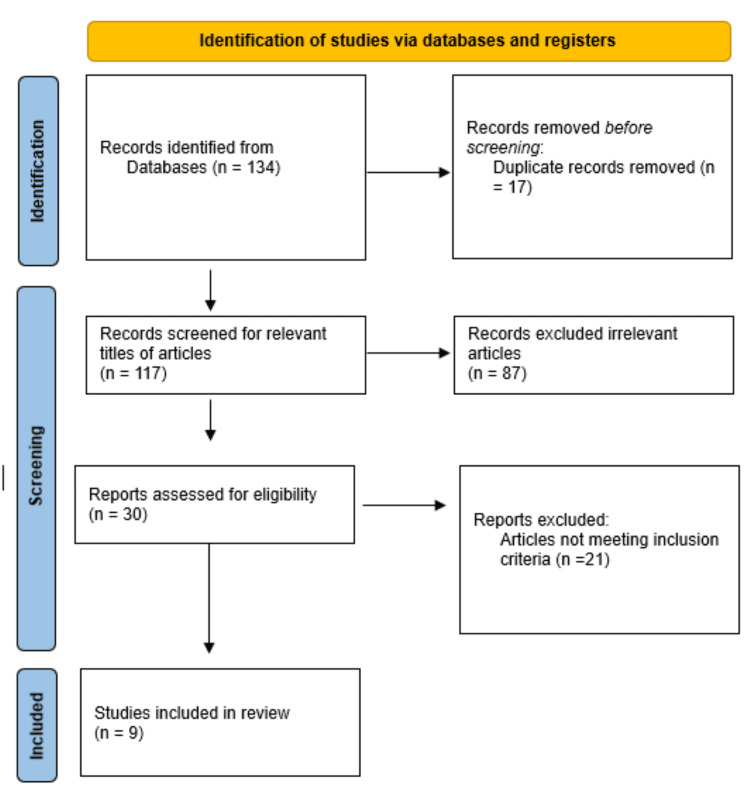
PRISMA flowchart PRISMA: Preferred Reporting Items for Systematic Reviews and Meta-Analyses

A search was conducted on electronic databases (PubMed, Google Scholar) from September 2012 to October 2023 using the search phrases "cholecystectomy" and "Diet post-cholecystectomy" in the abstract or title. The publication type, language restrictions, and study design criteria were used during the search. We removed documents that were duplicates. Randomized controlled trials (RCTs), non-randomized trials, and studies that examined the impact of any dietary factor in post-cholecystectomy patients, published in English from peer-reviewed journals, were included in the study. Studies from non-peer-reviewed journals and those unrelated to dietary factors were excluded. Numerous study designs were used, such as experimental studies, RCTs, and literature reviews. Following a preliminary investigation, 134 articles were found in the search database. We then eliminated 17 duplicate articles and excluded 87 articles that were irrelevant to the topic. After reviewing the full text of 30 articles, we excluded 21 because they did not meet the inclusion criteria. Nine articles were included in the final review. Figure 1 shows a summary of the selected publications based on the Preferred Reporting Items for Systematic Reviews and Meta-Analyses (PRISMA) guidelines.

Impact of dietary factors on cholecystectomy

The summary of articles reviewed for the study is mentioned in Table 1.

**Table 1:** Summary of articles reviewed for the impact of dietary factors on cholecystectomy GIQLI: gastrointestinal quality of life index; HDL-C: high-density lipoprotein cholesterol; BMI: body mass index; CHO: oral carbohydrate supplementation; VLCD: very low-calorie diet

Sr. no	Author and year	Participants	Intervention	Outcome	Conclusion
1	Blasco et al.[16]	83 patients older than 18 and had experienced biliary colic or complications from cholelithiasis	Patients were divided into four groups according to the dietary modifications given. Group 0: a very low-fat diet, Group 1: a low-fat diet, Group 2: a diet with a normal amount of fat, and Group 3: a diet rich in fat.	Comorbidities, surgery type, and demographic details were evaluated. Questions regarding symptoms and diet were asked prior to surgery, in person one month after surgery, and again six months after surgery as part of the examination. A questionnaire called the GIQLI has been used.	The study's primary findings showed that independent of dietary fat intake, the GIQLI score overall and the scores of the majority of the dimensions significantly increased one month and six months after surgery compared to baseline. After surgery, over 50% of patients reported changes in their bowel habits, and two-thirds reported persistent soft stools or diarrhea six months later. Dietary habits did not play a significant role, as half of the patients reported eating less fat after surgery, while the other half reported starting to consume a normal amount of fat six months post-surgery. Patient’s recommended low-fat diets do not appear to have an impact on how well their post-cholecystectomy symptoms are improving.
2	Shin et al.[17]	59 patients who underwent laparoscopic cholecystectomy	Patients were examined for experiencing symptoms like abdominal pain, dyspepsia, functional constipation, and diarrhea shortly after laparoscopic cholecystectomy and three months later.	Anthropometric measurements accompanied pre-operative and post-operative ultrasound evaluations to measure BMI. Serum biochemical examinations with automated tests were performed for a blood count and liver function test utilizing advanced automated systems. They provided a comprehensive understanding of the patient’s condition before and after the surgery. Patients also provided information on how frequently they had eaten each meal over the previous year. The absorption pattern was classified into 10 groups ranging from a few times a day to never/seldom daily. However, 24 hours post laparoscopic cholecystectomy was reviewed for dietary status.	Post-cholecystectomy syndrome risk correlated well with animal protein and cholesterol and eggs, positively and vegetable negatively intake three months post-laparoscopic cholecystectomy among the patients. Meanwhile, symptomatic patients ingested more bread at breakfast. Asymptomatic patients, on the contrary, preferred rice use at the same meal time. There is no significant immediate post-cholecystectomy relationship between the post-cholecystectomy syndrome and the patient's diet.
3	de Menezes et al. [18]	97 individuals with cholelithiasis planned for laparoscopic cholecystectomy	After surgery, Group I would consume a low-fat diet, and Group II would consume a regular diet.	Symptomatic examination was done after 15 days.	The findings of the study stated that a low-fat diet was not effective in the prevention of gastrointestinal symptoms, especially among the patients who were asymptomatic before the surgery. Therefore, a low-fat diet is not needed. Hence, the surgeons should evaluate the conditions and the patients' clinal status when adjusting their diet.
4	Öztepe et al. [19]	76 patients who underwent laparoscopic cholecystectomy	Patients were evaluated on the basis of food they consumed in the post-operative period.	First, a comprehensive descriptive characteristics form with 27 questions that gathered data on the majority of demographics and other related details were developed for the comprehensive examination. Second, the nutritional habit diagnosis form, consisting of 15 questions, was based on existing research and examined the physiological problems caused by specific foods after laparoscopic cholecystectomy. It contained questions on the consumption of 17 specific foods and queries about the symptoms experienced by participants.	The study found that symptoms increased with the intake of processed meats, full-fat cheese, certain vegetables, fruits, snack foods, sauces, and fried fatty foods post-laparoscopic cholecystectomy. This highlights the importance of evaluating pre-operative dietary habits and providing post-operative dietary guidance, particularly regarding foods that may worsen symptoms. Nurses' training in educating patients on these matters is crucial. Top of Form
5	Goodarzi et al. [20]	70 individuals undergoing cholecystectomy	The specific intervention was not given. Daily intake and composition of food were evaluated.	Before and one-month post-cholecystectomy, various variables, including weight, height, BMI, triglycerides, total cholesterol, low-density lipoprotein, HDL-C, and daily intake of calories, fat, protein, carbohydrates, fiber, saturated and unsaturated fat were examined.	The study showed that a reduction in the blood level of HDL-C after cholecystectomy can be observed in the first 30 days after surgery. In all other cases, several biomarkers, such as lipoproteins, weight, BMI, and dietary intake, showed no significant difference. It suggests the importance of observing similar parameters at several key time intervals in the early postoperative period. Only minimal modification in the diet between cholecystectomy and post-cholecystectomy cholesterol and serum lipoprotein levels can explain the apparent invariability before and after surgery. Regarding energy, fat, protein, carbohydrate, fiber, and saturated and unsaturated fat intake before and after the surgery, no discernible changes were found.
6	Yildiz et al. [21]	60 patients undergoing cholecystectomy, divided into two groups	While the Control group observed regular fasting, the Study group's patients received CHO in the evening before surgery and two to three hours before anesthesia.	The patients used a visual analog scale to rate their subjective feelings on five separate instances, which included weakness, thirst, hunger, malaise, fatigue, nausea, and difficulty concentrating.	In addition to lowering pre-operative malaise, thirst, hunger, and weakness, CHO preparation led to lower gastric volumes and higher gastric pH values, which in turn reduced perioperative rates of anxiety and depression in patients undergoing laparoscopic cholecystectomy under general anesthesia. As a result, it is safe to administer nonparticulate fluids high in oral carbohydrates prior to anesthesia.
7	Yazdankhah et al. [22]	48 individuals acquiring elective surgery for cholelithiasis symptoms	A week prior to surgery, patients were instructed to record every detail of their daily meals for 72 hours. A nutritionist then used NutriBase software to record and analyze these forms, allowing for the recording of the amount of energy, protein, fat, and carbohydrates consumed prior to surgery. Four and six months following the procedures, the patients were monitored.	Triglycerides and total cholesterol were taken from the patient's file.	Here, they demonstrated how patients will gain weight if their diets contain more fat and less protein, which could lead to weight gain and increased energy consumption. An increase in fat consumption following surgery was the primary cause of the weight gain and worsening of the lipid profile. A dietary consultation to control fat intake soon after surgery would enhance the prognosis of the patient following a cholecystectomy.
8	Burnand et al. [23]	46 patients undergoing laparoscopic cholecystectomy divided into two groups	One group was given a low-calorie diet, and another group was advised for VLCD. Participants who were placed in the low-calorie arm received a diet sheet to adhere to. The VLCD consisted of a two-week calorie-restricted diet with an 800 kcal daily goal for total caloric intake. Advice from dietitians was available to both study arms. Before the procedure, every patient was required to fill out an extensive food survey for the two weeks before.	Operational time, calculated from the beginning of the incision to the conclusion of skin closure, served as the study's main outcome measure. The weight changes from the pre-operative clinic to the procedure day, length of stay, post-operative complications, and day-case rates were the secondary outcomes. The surgeon evaluated the procedure's perceived difficulty at the conclusion of each operation.	Pre-operative weight loss was significantly greater with the VLCD, and it was well tolerated. In obese patients, a two-week VLCD before an elective laparoscopic cholecystectomy is safe and well tolerated and has been demonstrated to lower pre-operative weight and surgical time significantly.
9	Barth et al. [24]	60 patients undergoing partial hepatectomy with a body mass index of 25 kg/m².	Either no special diet for one week prior to liver resection or an 800-kcal, 20 g fat, and 70 g protein diet.	Intraoperative blood loss was the study's main outcome measure. Measures of secondary outcomes include evaluation of the intraoperative liver mobility, amount of blood transfusion, duration of stay, death, steatosis, steatohepatitis, and amount of glycogen in hepatocytes.	Short-term, low-fat, low-calorie diets dramatically reduce bleeding and facilitate easier liver manipulation during hepatic surgery.

Both biliary and non-biliary disorders are included in the diverse group of gastrointestinal symptoms known as PCS, which arises after cholecystectomy. After surgery, symptoms such as nausea, flatulence, acidity, diarrhea, constipation, abdominal distension, and dyspepsia may go away or reappear. After a cholecystectomy, a low-fat diet has not always been advised, and there are no established recommendations for nutrition following surgery [25-27]. In a study conducted by Blasco et al., dietary changes were made to target the fat content in 83 patients who had cholecystectomy. The study found that there was no discernible difference in the impact of low-fat content on the rate of improvement of post-operative symptoms [16]. As the GB is an organ of storage rather than bile production, there is currently no good reason to restrict a patient's diet during the post-operative phase of a cholecystectomy. However, many experts advise exercising caution and limiting lipid intake in the initial weeks following surgery, aiming to lower the risk of developing symptoms of PCS [28,29].

On the other hand, de Menezes et al. conducted a study on post-cholecystectomy patients, recommending a low-fat diet. They concluded that a low-fat diet was not associated with gastrointestinal symptoms. According to the authors, diets should be customized according to individual needs [18]. The debate among surgeons about whether these patients should follow a low-fat diet following surgery remains a cause for debate. There are currently no scientific studies that demonstrate whether a low-fat diet is effective after a cholecystectomy. Shin et al. also evaluated the routine diet of patients who went through cholecystectomy and found that the risk for PCS was increased with animal protein and cholesterol-rich food and found a relationship between the symptoms and dietary factors [17].

There are no established recommendations for nutrition following a cholecystectomy in this regard. However, the literature indicates that dietary modifications, such as reducing fat intake, limiting overindulgence in meals, increasing fiber intake, and avoiding alcohol, carbonated and caffeinated drinks, chocolate, citrus foods, fruit juices, coffee, vinegar sauce, onions, tomatoes, and spicy foods, can help prevent reflux [30,22]. Öztepe et al. found that after laparoscopic cholecystectomy, symptoms worsened with the consumption of processed meats, full-fat cheese, some fruits and vegetables, snack foods, sauces, and fried, high-fat foods. This emphasizes how crucial it is to assess dietary practices prior to surgery and offer post-operative dietary advice [19].

Individuals who have dyslipidemia are more likely to develop gallstone disease, which can especially occur in those whose triglyceride metabolism is impaired. A well-established pathophysiologic component connected to obesity. However, the GB does more than just store and concentrate BAs; it may also have an impact on how nutrients, particularly lipids, are metabolized, which, in turn, affects how much of these nutrients are present in the blood. As a result, the removal of this organ is likely to cause metabolic changes that could change the biomarkers' serum levels [31-33]. Goodarzi et al. did a study on 70 patients who evaluated the daily dietary consumption, and the study revealed that by the 30th day following surgery, cholecystectomy lowers high-density lipoprotein cholesterol (HDL-C) levels; however, lipoproteins, weight, and BMI did not change substantially. Furthermore, minor dietary changes may be the cause of the stability of lipoprotein serum levels both before and after a cholecystectomy [20].

Discussion

The findings from the reviewed literature show that there are several points regarding the role of dietary measures in the alleviation of post-cholecystectomy symptoms and outcomes. Firstly, the findings from the conducted studies suggest that although dietary changes are a common recommendation, these measures are not sufficiently supported by evidence when it comes to alleviating symptoms and improving outcomes post-cholecystectomy. More specifically, as evidenced by Blasco et al. and de Menezes et al., there is no significant relationship between fat intake and post-surgical symptom improvement due to the variation in the amount of fat consumed by the patients. Veritably, it necessitates a more personalized approach to dieting adjusted for the individual needs of the patient [16,18].

Secondly, the studies reveal the need for pre-operative dietary assessment and post-operative dietary suggestions for symptoms and outcomes. Studies found that subjects who consumed particular foods, such as processed meat and fried fatty foods, had exacerbated symptoms after cholecystectomy. Research on mice showed that long-term diet intervention and probiotics might alleviate the risk of post-cholecystectomy by alleviating gut microbiota and BA disorders. The results of the studies indicate that establishing counseling on diets before and after the surgical procedure could enhance patient quality of life and outcomes.

The recommendation for the approach is toward personalized treatment of post-cholecystectomy symptoms, which suggests a holistic approach that takes into account the pre-operative factors and post-operative diet. They further argue the need for additional studies to better comprehend how patients' characteristics interact with dietary habits toward post-operative effects in order to ultimately enhance patient care and life after cholecystectomy.

## Conclusions

The purpose of this review was to look at the various effects of different dietary factors on patients undergoing cholecystectomy, how they affect their overall health after surgery, and how they contribute to symptoms of PCS. After a cholecystectomy, the body's ability to digest lipids is somewhat reduced due to the absence of the GB. Some individuals may experience mild discomfort or alterations in bowel patterns, especially after consuming high-fat meals. The studies revealed that there was not a huge impact of fat content in food on post-cholecystectomy symptoms. However, individuals who consumed particular foods, such as processed meat and fried fatty foods, had exacerbated symptoms after cholecystectomy. Although the role of dietary interventions in the management of post-cholecystectomy symptoms and outcomes remains complex and multifactorial, the results from these investigations underscore the need for personalized dietary counseling that suits a particular person’s requirements. Further studies are still required to understand the precise food factors that might affect post-surgical symptoms and outcomes, as well as to develop tailored measures to enhance patient care and long-term prognosis after undergoing cholecystectomy.

## References

[1] Shaffer EA (2000). Review article: control of gall-bladder motor function. Aliment Pharmacol Ther.

[2] Maldonado-Valderrama J, Wilde P, Macierzanka A, Mackie A (2011). The role of bile salts in digestion. Adv Colloid Interface Sci.

[3] Bertolini A, Fiorotto R, Strazzabosco M (2022). Bile acids and their receptors: modulators and therapeutic targets in liver inflammation. Semin Immunopathol.

[4] Xu F, Yu Z, Liu Y (2023). A high-fat, high-cholesterol diet promotes intestinal inflammation by exacerbating gut microbiome dysbiosis and bile acid disorders in cholecystectomy. Nutrients.

[5] Zdanowicz K, Daniluk J, Lebensztejn DM, Daniluk U (2022). The etiology of cholelithiasis in children and adolescents-a literature review. Int J Mol Sci.

[6] Barie PS, Eachempati SR (2010). Acute acalculous cholecystitis. Gastroenterol Clin North Am.

[7] Fu Y, Pang L, Dai W, Wu S, Kong J (2022). Advances in the study of acute acalculous cholecystitis: a comprehensive review. Dig Dis.

[8] Vitetta L, Sali A, Little P, Mrazek L (2000). Gallstones and gall bladder carcinoma. Aust N Z J Surg.

[9] Strasberg SM (2019). A three-step conceptual roadmap for avoiding bile duct injury in laparoscopic cholecystectomy: an invited perspective review. J Hepatobiliary Pancreat Sci.

[10] Coccolini F, Catena F, Pisano M (2015). Open versus laparoscopic cholecystectomy in acute cholecystitis. Systematic review and meta-analysis. Int J Surg.

[11] Kyriacou E (1999). Carcinoma of the gall-bladder. J Gastroenterol Hepatol.

[12] Sato Y, Atarashi K, Plichta DR (2021). Novel bile acid biosynthetic pathways are enriched in the microbiome of centenarians. Nature.

[13] Magnano San Lio R, Barchitta M, Maugeri A, Quartarone S, Basile G, Agodi A (2022). Pre-operative risk factors for conversion from laparoscopic to open cholecystectomy: a systematic review and meta-analysis. Int J Environ Res Public Health.

[14] Marcason W (2014). What medical nutrition therapy guideline is recommended post-cholecystectomy?. J Acad Nutr Diet.

[15] Yueh TP, Chen FY, Lin TE, Chuang MT (2014). Diarrhea after laparoscopic cholecystectomy: associated factors and predictors. Asian J Surg.

[16] Blasco Y, Muñante M, Gómez FL, Jovell E, Bernad LM (2020). Low-fat diet after cholecystectomy: should it be systematically recommended?. Cir Esp Engl Ed.

[17] Shin Y, Choi D, Lee KG, Choi HS, Park Y (2018). Association between dietary intake and postlaparoscopic cholecystectomic symptoms in patients with gallbladder disease. Korean J Intern Med.

[18] de Menezes HL, Fireman PA, Wanderley VE, de Menconça AM, Bispo RK, Reis MR (2013). Randomized study for assessment of hypolipidic diet in digestive symptoms immediately following laparoscopic cholecystectomy. Rev Col Bras Cir.

[19] Öztepe K, Çavdar İ, Aksakal N (2023). Evaluation of change in nutrition after laparoscopic cholecystectomy. J Health Sci Med.

[20] Goodarzi R, Saedisomeolia A, Moghadam EF, Abbasi A, Sianaki A, Seaf Z (2017). Evaluation of the serum lipid profile and dietary intake in patients undergoing cholecystectomy. A v Stud Biol.

[21] Yildiz H, Gunal SE, Yilmaz G, Yucel S (2013). Oral carbohydrate supplementation reduces preoperative discomfort in laparoscopic cholecystectomy. J Invest Surg.

[22] Yazdankhah KA, Yaghoobi NA Jr, Nazari M (2012). Measuring the rate of weight gain and the influential role of diet in patients undergoing elective laparoscopic cholecystectomy: a 6-month follow-up study. Int J Food Sci Nutr.

[23] Burnand KM, Lahiri RP, Burr N, Jansen van Rensburg L, Lewis MP (2016). A randomised, single blinded trial, assessing the effect of a two week preoperative very low calorie diet on laparoscopic cholecystectomy in obese patients. HPB (Oxford).

[24] Barth RJ Jr, Mills JB, Suriawinata AA (2019). Short-term pre-operative diet decreases bleeding after partial hepatectomy: results from a multi-institutional randomized controlled trial. Ann Surg.

[25] Matsudaira S, Fukumoto T, Yarita A, Hamada J, Hisada M, Fukushima J, Kawarabayashi N (2020). A patient with cystic duct remnant calculus treated by laparoscopic surgery combined with near-infrared fluorescence cholangiography. Surg Case Rep.

[26] Filip M, Saftoiu A, Popescu C, Gheonea DI, Iordache S, Sandulescu L, Ciurea T (2009). Postcholecystectomy syndrome - an algorithmic approach. J Gastrointestin Liver Dis.

[27] Lublin M, Crawford DL, Hiatt JR, Phillips EH (2004). Symptoms before and after laparoscopic cholecystectomy for gallstones. Am Surg.

[28] Borly L, Anderson IB, Bardram L (1999). Preoperative prediction model of outcome after cholecystectomy for symptomatic gallstones. Scand J Gastroenterol.

[29] Luman W, Adams WH, Nixon SN, Mcintyre IM, Hamer-Hodges D, Wilson G, Palmer KR (1996). Incidence of persistent symptoms after laparoscopic cholecystectomy: a prospective study. Gut.

[30] Ozyel B, Malyali N (2020). Importance of dietary consultation after cholecystectomy: pre- and post-cholecystectomy nutritional status, dietary habits and anthropometric measures of patients. Proc Nutr Soc.

[31] Staels B, Handelsman Y, Fonseca V (2010). Bile acid sequestrants for lipid and glucose control. Curr Diab Rep.

[32] Juvonen T, Kervinen K, Kairaluoma MI, Kesäniemi YA (1995). Effect of cholecystectomy on plasma lipid and lipoprotein levels. Hepatogastroenterology.

[33] Nervi F, Arrese M (2013). Cholecystectomy and NAFLD: does gallbladder removal have metabolic consequences?. Am J Gastroenterol.

